# Double-gate thin film transistor with suspended-gate applicable to tactile force sensor

**DOI:** 10.1186/s40580-020-00240-9

**Published:** 2020-09-15

**Authors:** An Hoang-Thuy Nguyen, Manh-Cuong Nguyen, Seongyong Cho, Anh-Duy Nguyen, Hyewon Kim, Yeongcheol Seok, Jiyeon Yoon, Rino Choi

**Affiliations:** Department of Materials Science and Engineering, Inha University, Incheon, 22212 South Korea

**Keywords:** Mechanical sensors, Air gap, Membrane gate, Photoresist sacrifice, Tactile force sensor

## Abstract

This paper presents a straightforward, low-cost, and effective integration process for the fabrication of membrane gate thin film transistors (TFTs) with an air gap. The membrane gate TFT with an air gap can be used as the highly sensitive tactile force sensor. The suspended membrane gate with an air gap as the insulator layer is formed by multiple photolithography steps and photoresist sacrificial layers. The viscosity of the photoresist and the spin speed was used to modify the thickness of the air gap during the coating process. The tactile force was measured by monitoring the drain current of the TFT as the force changed the thickness of the air gap. The sensitivity of the devices was enhanced by an optimal gate size and low Young’s modulus of the gate material. This simple process has the potential for the production of small, versatile, and highly sensitive sensors.

## Introduction

Recently, a range of microelectromechanical systems (MEMS) have been proposed and fabricated for a range of applications. MEMS allows the miniaturization of mechanical device structures, such as sensors, transducers, and some microelectronics [1–4]. The convergence of a complementary metal-oxide-semiconductor (CMOS) and a microelectrochemical system to produce a suspended-gate metal-oxide-semiconductor field-effect transistor (MOSFET) with an air gap was proposed as a methodology to fabricate a new generation of sensors, with some spectacular results reported [5, 6]. A suspended-gate air gap MOSFET built on a Si channel has been reported [7, 8] but the optical and mechanical properties of silicon have limited their applications in the flexible, transparent, and wearable sensors compares to the other device structures [9, 10]. For that application, suspended-gate thin-film transistors (TFTs) with organic or inorganic semiconductors have attracted considerable attention. Zang et al, reported ultra-sensitive pressure detection sensors using organic TFTs with a spectacular suspended-gate structure with an air gap as the dielectric layer [11]. Mannsfeld et al. presented a capacitive pressure sensor with a micro-structured PDMS as the dielectric layers for an organic single-crystal transistor that exhibited unprecedented sensitivity and short response times [12]. Zang et al. reported a suspended composite gate structure with Ag nanowires, Fe_3_O_4_, and PDMS for organic transistors as flexible magnetic sensors [13]. Despite these advantages, such as great sensitivity and rapid response time in recording pressure, acoustic wave realization, and magnetic fields, their device structures are incompatible with the conventional CMOS process. In addition, organic semiconductors still have issues in conductivity and stability for channel materials. Therefore, CMOS process-compatible inorganic oxide semiconductors are needed to replace their organic counterparts as a TFT channel material.

Since the high performance of an amorphous indium-gallium-zinc-oxide (IGZO) semiconductor for TFTs was presented [14], several studies have reported higher mobility, smaller subthreshold-swing, and better stability of this oxide semiconductor. Based on the enhancement of performance, IGZO thin films have replaced amorphous silicon (a-Si) in flat panel display applications. The IGZO semiconductor has advantages over organic semiconductors in terms of the optical-electric, low deposition temperature, low-cost, bendable and invisible devices, and compatibility with conventional fabrication methods [15]. On the other hand, few studies have used IGZO channel TFTs for tactile force sensors with a low-temperature process. These studies used organic or piezoelectric materials that cannot be implemented easily in conventional microfabrication. Moreover, the number of process steps is large because they combine different device structures.

This paper reports a suspended-gate TFT structure with an air gap insulator and IZGO channel for tactile force sensor applications. The devices were first built on a SiO_2_/Si substrate by microfabrication, including multiple ultraviolet (UV) light exposure steps for the dual-gate structure. An alumina layer was deposited by a spin coating process as the protective dielectric layer, which prevents damage from the gate collapse  [16]. The air gap acts as an insulator layer for TFTs and improves the sensitivity by allowing a movable suspended gate. The air gap thickness changes when a stress is applied to the gate, which leads to varying electrostatic capacitance values on the channel and exchanges into different drain currents in TFTs. The tactile pressure quantity and the sensitivity can be calculated from the measured drain current.

## Experimental

**Fig. 1:**
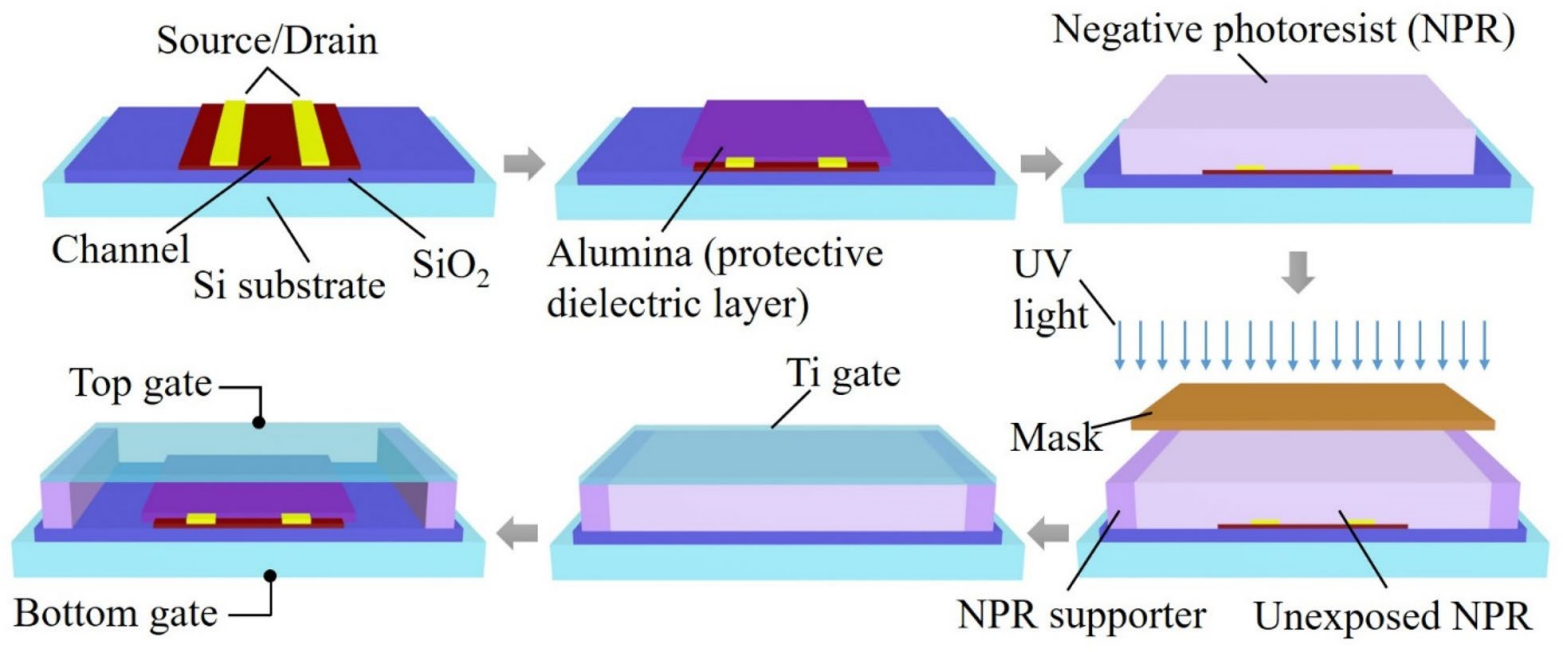
Schematic diagram of process flow for IGZO suspended-gate TFT with an air gap

Figure 1 presents a schematic process flow for the dual-gate TFTs with the IGZO channel. The 30 nm IGZO channel was deposited by radio frequency sputtering at room temperature and annealed at 400 °C for one hour in air ambient on SiO_2_ (100 nm)/Si substrate for the bottom gate. A 50 nm tungsten (W) layer for the source and drain was patterned by a lift-off process and deposited by direct current (DC) magnetron sputtering. A 20 nm thick alumina (Al_2_O_3_) layer, as the protective dielectric layer, was coated by a spin coating solution process at a speed of 3000 rpm for 20 s. Aluminum nitrate nonahydrate (Sigma-Aldrich, 99.997% trace metals basis) and 2-methoxyethanol (Sigma-Aldrich, anhydrous 99.8%) as precursors (a 0.8 M solution was prepared and stirred for 5 h) were used for the solution. The samples were annealed at 250 °C for 1 h in air after coating. The alumina layer was patterned to open the source and drain contacts. A negative photoresist (NPR) layer as the supporter was coated and exposed to ultraviolet light for the air gap layer without a developing step. A 500 nm Ti was deposited on top of the NPR layer by DC magnetron sputter for the top gate, which is a movable structure. The gate was patterned by one more photolithography step with a positive photoresist. After the developing step, the remaining photoresist was exposed once more time to UV light before the etching stage. Ti was etched in HF:H_2_O_2_:H_2_O = 1:1:20 solution for 20 s. The suspended-gate was formed in the developer solution by removing the negative and positive photoresist above and below the Ti gate. The thickness of each layer was analyzed by X-ray reflectivity (XRR) measurement. The structure of the device was examined by scanning electron microscopy (SEM). The transfer current was measured using an Agilent 4155C semiconductor analyzer at room temperature in a dark chamber with and without pressure on the gate.

## Results and discussion

**Fig. 2:**
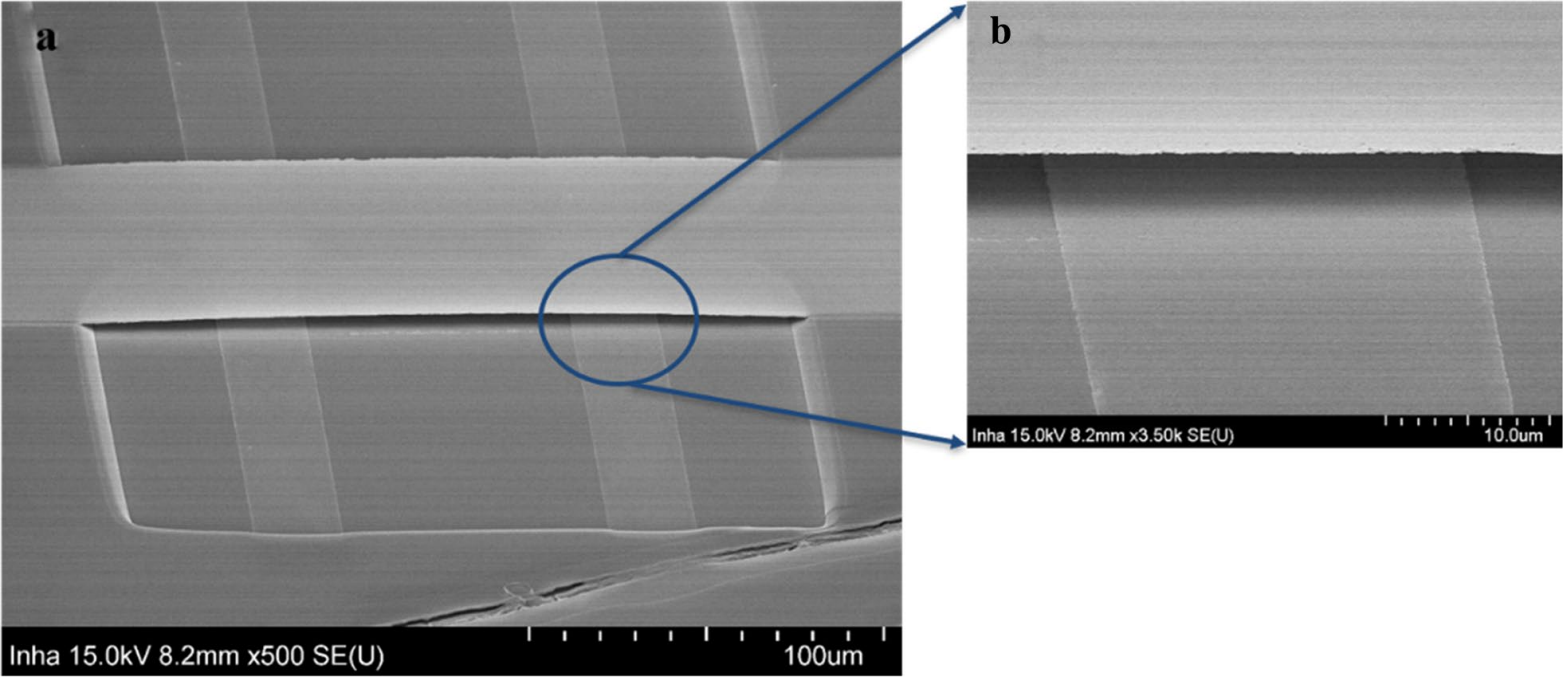
**a** SEM image of Ti suspended-gate structure with a 200 μm-long and 50 μm-wide. **b** Closer look of the inserted blue round area

SEM confirmed the formation of a Ti suspended-gate, 200 μm in length and 50 μm in width, over the channel, as shown in Fig. 2. In the process, the NPR was a supporter to prop the Ti gate and was used to determine the air gap thickness. The thickness of the NPR was controlled by adjusting the speed of the spin coating step. In this device, the air gap was found to be approximately 5 μm. After the vacuum drying step, there was no photoresist or water observed under the Ti gate, proving that the process can release the thin metal film as a suspended-gate for TFT devices.

**Fig. 3:**
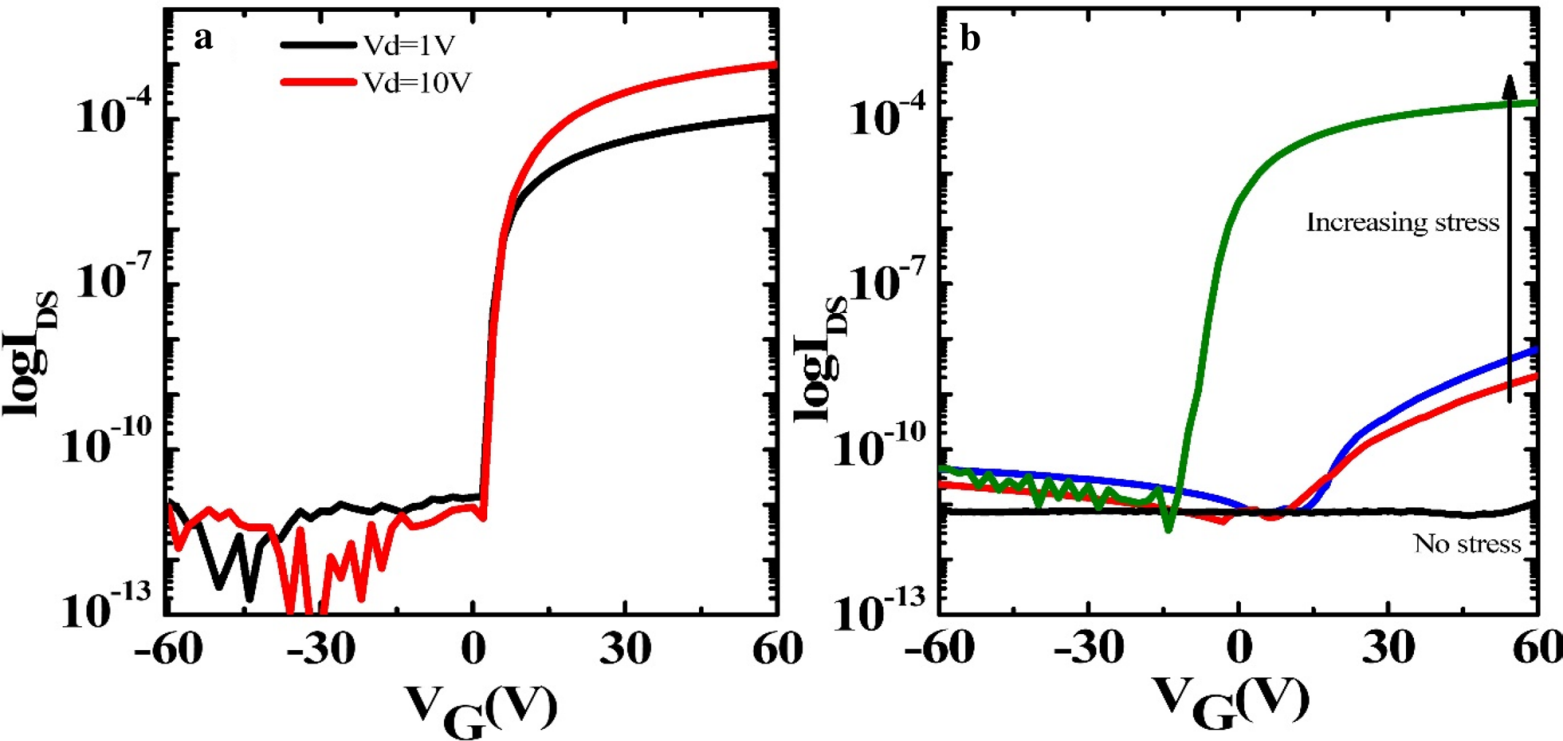
Transfer curves of the IGZO back gate TFTs with **a** 100 nm SiO_2_ as insulator layer at V_d_ = 1 and 10 V and **b** suspended-gate including the 20 nm alumina as the protective dielectric and an air gap

Figure 3 presents the transfer curves that measure from the back gate structure with a SiO_2_ insulator and top gate structure with an Al_2_O_3_ insulator. The bottom gate was used to confirm that the channel is working after the entire process. For the back gate structure, the device had the W/L = 400/50 and the drain current (I_ds_) was measured with the gate voltage (V_g_) scanned from -60 to 60 V at a drain voltage V_d_ = 1 V and 10 V. The field-effect mobility of the back gate device was approximately 8.7 cm^2^V^−1^s^−1^. The back-gate structure was fabricated to calculate the saturation mobility of IGZO at high V_d_. For the top gate structure, the I_d_ was swept with V_g_ from − 60 to 60 V at V_d_ = 1 V with the W/L = 50/50 based on the Ti gate size. The pressure was placed on the surface of the Ti suspended-gate using an insulated probe tip at the top to increase the contact area and prevent the current to the tip. Without the application of stress to the top gate, there was no drain current passing through the channel, even though the gate voltage was increased up to 60 V. The drain current increased gradually by applying pressure to the tip. The threshold voltages were also shifted from 42 to 3.9 V. The entire enhancement in the electrical properties shows that the suspended-gate TFT with the IGZO channel can detect the diverse pressure. After pushing the gate with the tip and releasing it, the drain current did not return to the value before stressing due to damage from pressing the gate.

In this structure, the insulator layer for oxide TFTs involves the alumina protective dielectric. This prevents the suspended gate from coming into contact with the channel directly as excessive pressure is applied. Direct contact of the suspended gate and channel leads to the collapse of the gate and damage to the channel. The other insulator layer is the air gap, which has the rubber sensitivity role for the tactile force sensor. The pressure was applied using an insulated probe tip. The drain current in the saturation region of the suspended-gate TFT can be calculated from the following equation  [17]

1 $$I_{DS} = \frac{{W\mu_{sat} C_{t} }}{2L}\left( {V_{g} - V_{t} } \right)^{2}$$

where W and L are the channel width and length of TFT, $V_{g}$ and $V_{t}$ are the gate voltage and threshold voltage, respectively; $\mu_{sat}$ is the saturation mobility, and $C_{t}$ is the total capacitance of the insulator layers, which is expressed as  [17]:

2 $$\frac{1}{{C_{t} }} = \frac{1}{{C_{pd} }} + \frac{1}{{C_{gap} }}$$

where $C_{pd}$ and $C_{gap}$ are the protective dielectric (alumina) and air gap capacitance, respectively. $C_{gap}$ can be expressed as

3 $$C_{gap} = \frac{{\varepsilon_{air} \varepsilon_{0} A}}{{d_{gap} }}$$

where $\varepsilon_{air}$, $\varepsilon_{0}$, A, and d_gap_ are the relative dielectric constant of air, absolute dielectric constant, area of the gate of TFT, and air gap thickness, respectively. By changing d_gap_ with the pressure on the suspended gate, C_gap_ can be modulated, and I_ds_ would increase or decrease accordingly. The accurate displacements according to the air gap thicknesses from the transfer curves were calculated.

**Fig. 4:**
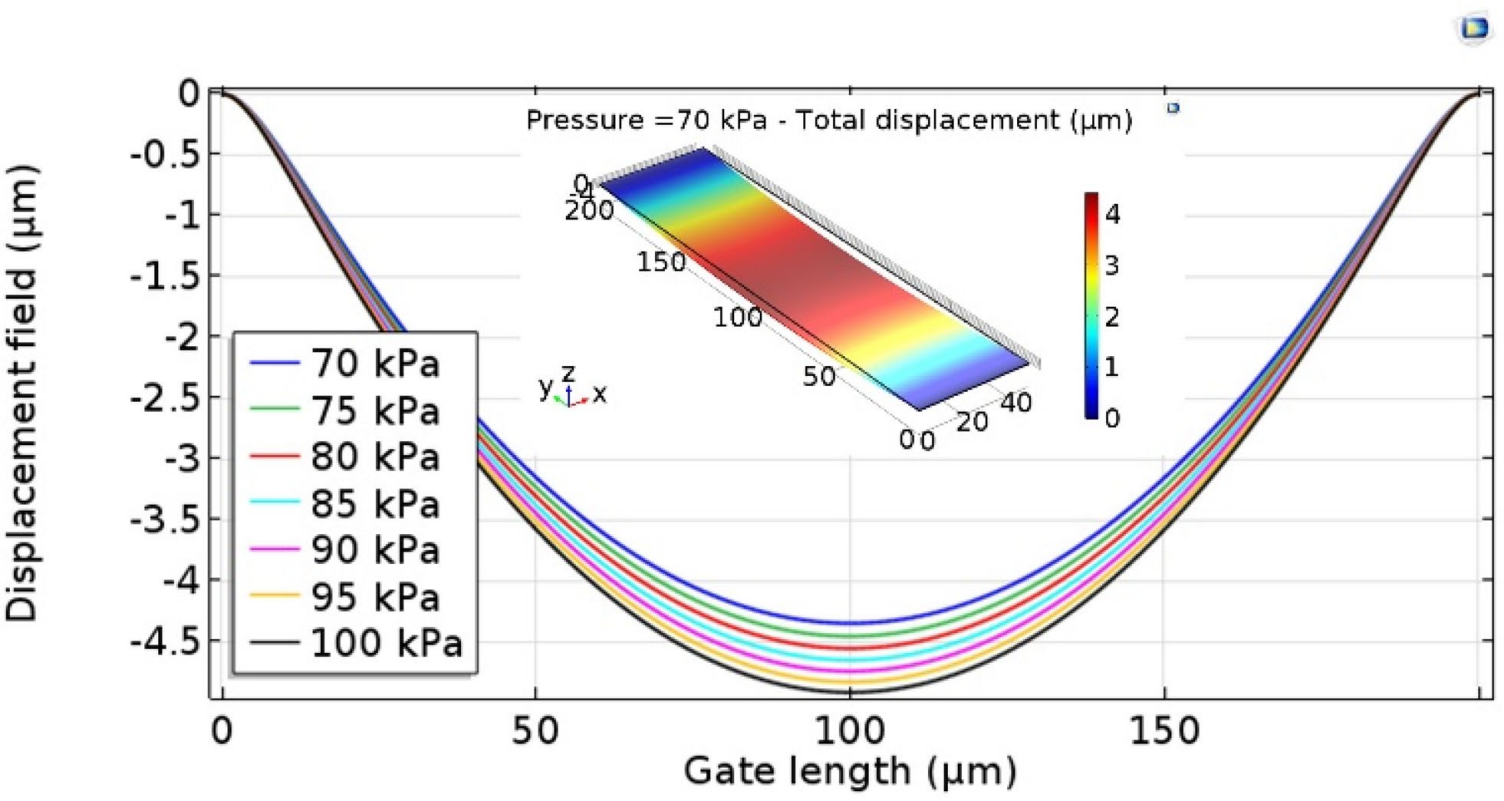
Displacement field profile of the movable gate for various applied stresses. The inset figure shows COMSOL simulation for the movable Ti gate with pressing 70 kPa on the surface

Figure 4 shows the displacement of the Ti gate simulated from the COMSOL simulation when the stress to the gate was varied from 75 to 100 kPa. In the simulation, the Ti gate has a matching size with a movable part in the practical device structure, and Young’s modulus, density, and Poisson’s ratio were 116 GPa, 4506 kg.m^−3^, and 0.3, respectively. The displacements in the middle of the gate increased from 4.5 to 5 μm as the pressure was increased. When the stress was higher than 100 kPa, the displacement became larger than the air gap thickness, 5 μm.

**Fig. 5:**
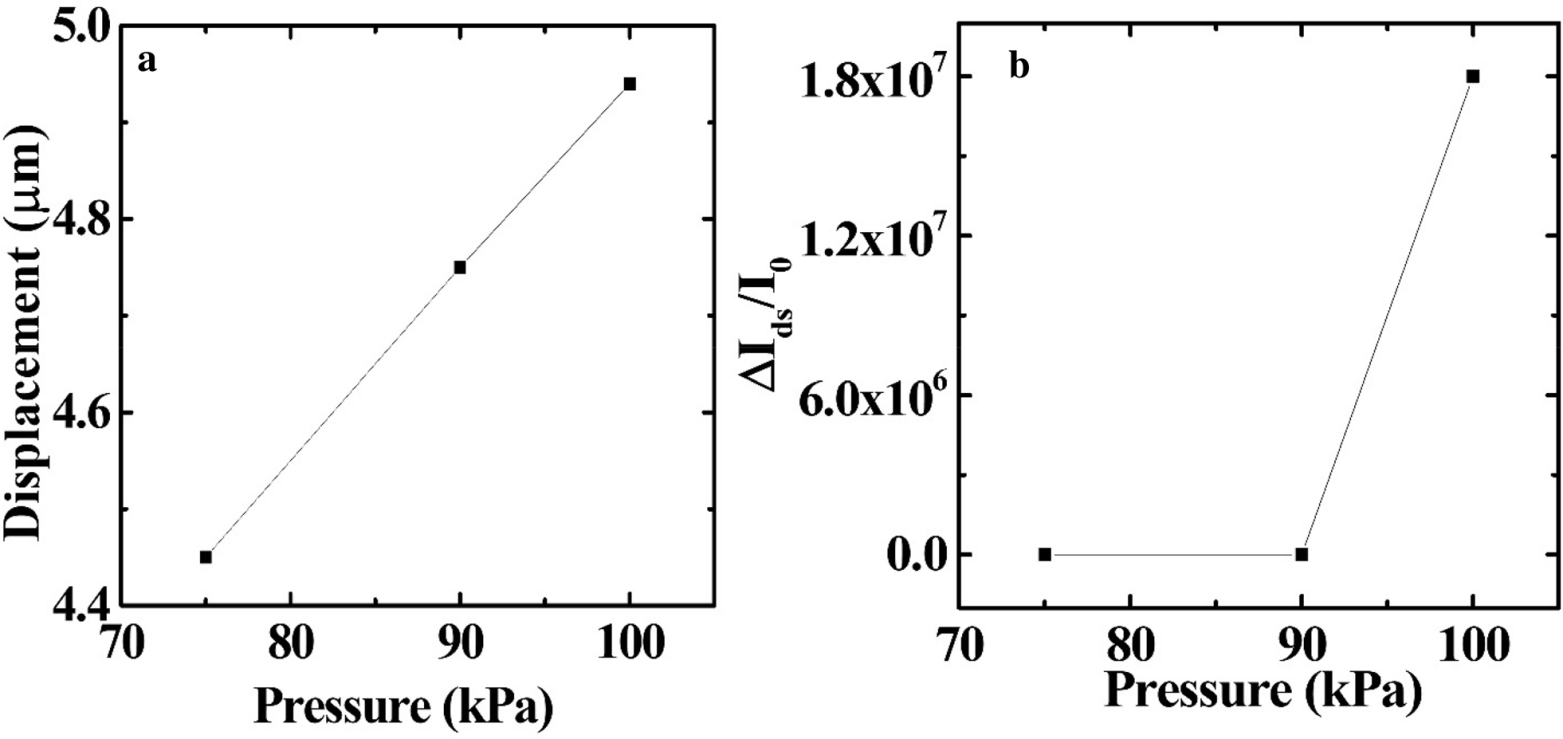
**a** Relationship between the pressure on the gate and displacement of the gate. **b** Sensitivity of 500 nm Ti suspended-gate at V_g_ = 60 V, V_d_ = 1 V

The applied stress corresponding to the displacement was extracted by matching the displacement from the simulation and calculation based on (3), as shown in Fig. 5a. Figure 5b shows the difference in the drain current ∆I_ds_/I_0_ (∆I_ds_ is the relative change in the drain current corresponding to the change in pressure loading ∆P and I_0_ is the initial current without pressure  [11]) under various pressures. The sensitivity of the devices was also calculated using the sensitivity, which was defined as S = (∆I_ds_/I_0_)/∆P. The highest sensitivity was observed at the largest applied pressure of 180 Pa^−1^.

The sensitivity acquired from the measured current curve showed high practicability for a range of applications, which overcomes the difficulties of the polymer dielectric. Because the air gap is used as the dielectric layer in a field-effect transistor, a higher sensitivity target was reached with the microsensor device. Despite the great sensitivity, this structure may have problems in the recovery of the suspended gate after removing the pressure because of the thicker Ti film thickness and the large gate size. This problem can be alleviated by covering one PDMS layer as a passivation layer and improving the resilience of the metal gate [18].

## Conclusions

A suspended-gate oxide TFT was fabricated using an IGZO channel for the tactile force sensor. Using the simple fab-compatible process with multi photolithography steps, a Ti suspended-gate, 200 μm in length and 50 μm in width, was built on the negative photoresist supporters. The characteristics of a tactile force sensor were demonstrated by combining with the low-temperature process for IGZO TFT. From the transfer curve of oxide TFT, the sensitivity was calculated to be 180 Pa^−1^ at V_d_ = 1 V and V_g_ = 60 V. The IGZO TFT with the air gap for the insulator layer enhanced the sensitivity of the sensor substantially.

## Data Availability

Not applicable.

## Abbreviations

MEMS: Microelectromechanical systems
CMOS: Complementary metal-oxide-semiconductor
MOSFETs: Metal-oxide-semiconductor field-effect transistors
TFTs: Thin-film transistors
PDMS: Polydimethylsiloxane
IGZO: Indium-gallium-zinc-oxide
a-Si: Amorphous silicon
UV: Ultraviolet
W: Tungsten
DC: Direct current
NPR: Negative photoresist
XRR: X-ray reflectivity
SEM: Scanning electron microscopy
